# Effect of mTOR inhibitors on sodium taurocholate cotransporting polypeptide (NTCP) function *in vitro*

**DOI:** 10.3389/fphar.2023.1147495

**Published:** 2023-03-22

**Authors:** Chitra Saran, Henry Ho, Paavo Honkakoski, Kim L. R. Brouwer

**Affiliations:** ^1^ Department of Pharmacology, UNC School of Medicine, University of North Carolina, Chapel Hill, NC, United States; ^2^ Division of Pharmacotherapy and Experimental Therapeutics, UNC Eshelman School of Pharmacy, University of North Carolina, Chapel Hill, NC, United States; ^3^ School of Pharmacy, University of Eastern Finland, Kuopio, Finland

**Keywords:** mTOR inhibitors, kinase inhibitors, NTCP, taurocholate, hepatitis virus, HuH-7 cells, Flp-In T-REx 293 cells, everolimus

## Abstract

The sodium taurocholate cotransporting polypeptide (NTCP; gene name *SLC10A1*) is the primary hepatic basolateral uptake transporter for conjugated bile acids and the entry receptor for the hepatitis B and D virus (HBV/HDV). Regulation of human NTCP remains a knowledge gap due to significant species differences in substrate and inhibitor selectivity and plasma membrane expression. In the present study, various kinase inhibitors were screened for inhibition of NTCP function and taurocholate (TCA) uptake using NTCP-transfected HuH-7 cells. This study identified everolimus, an mTOR inhibitor and macrocyclic immunosuppressive drug, as an NTCP inhibitor with modest potency (IC_50_ = 6.7–8.0 µM). Further investigation in differentiated HuH-7 cells expressing NTCP and NTCP-overexpressing Flp-In T-REx 293 cells revealed that the mechanism of action of everolimus on NTCP is direct inhibition and mTOR-independent. Structural analogs of everolimus inhibited NTCP-mediated TCA uptake, however, functional analogs did not affect NTCP-mediated TCA transport, providing further evidence for direct inhibition. This work contributes to the growing body of literature suggesting that NTCP-mediated bile acid uptake may be inhibited by macrocyclic peptides, which may be further exploited to develop novel medications against HBV/HDV.

## 1 Introduction

The sodium taurocholate cotransporting polypeptide (NTCP) protein is expressed exclusively on the basolateral membrane of hepatocytes (Hagenbuch and Meier, 1994). As the primary bile acid uptake transporter in hepatocytes, NTCP plays a critical role in bile acid circulation and homeostasis (Appelman et al., 2021). NTCP-mediated bile acid transport accounts for >80% of conjugated bile acid uptake and <50% of unconjugated bile acid uptake into human hepatocytes (Shitara et al., 2003; Kosters and Karpen, 2008). NTCP transport is strictly sodium-dependent; in addition to bile acids, NTCP transports steroid hormones, thyroid hormones, and some drugs (e.g., rosuvastatin) (Claro da Silva et al., 2013; Anwer and Stieger, 2014). Of relevance to infectious diseases, NTCP acts as the entry receptor for hepatitis B virus (HBV) and hepatitis D virus (HDV), which infect nearly 350 million people globally (Yan et al., 2012). Significant efforts to identify specific NTCP inhibitors led to the discovery of Bulevirtide or Myrcludex B, a synthetic, myristoylated peptide that mimics amino acids 2–48 of a high-affinity NTCP binding motif of HBV (preS1 domain) (Schulze et al., 2010; Meier et al., 2013). Myrcludex B inhibits the binding of NTCP to the HBV preS1 domain with high efficacy and is in clinical trials for HBV and HDV treatment (Donkers et al., 2019). Cyclosporin A has been shown to inhibit NTCP function and also inhibits HBV entry into hepatocytes (Watashi et al., 2014; Yuen and Lai, 2015).

Despite >73% sequence identity between human NTCP and mouse/rat Ntcp, there are key species differences in transport characteristics (Geyer et al., 2006; Kosters and Karpen, 2008; Anwer and Stieger, 2014). For example, rosuvastatin is transported by human NTCP, but not by rat Ntcp (Ho et al., 2006). In addition to species differences in rosuvastatin transport, bosentan inhibits rat Ntcp more potently than human NTCP (Leslie et al., 2007). Taurolithocholate (TLCA) decreased Ntcp in rat plasma membranes but did not impact NTCP in human plasma membranes, while nitric oxide decreased NTCP in human plasma membranes but did not alter Ntcp in rat plasma membranes (Anwer and Stieger, 2014). Unlike human NTCP, mouse Ntcp does not support HBV or HDV infection despite binding to the HBV preS1 domain (Ni et al., 2014). Considering the species differences in NTCP/Ntcp function, the primary goal of this study was to develop human NTCP-expressing cell systems using HuH-7 cells and Flp-In^™^ T-REx^™^ 293 cells to identify novel NTCP inhibitors. The Flp-In^™^ T-REx^™^ 293 cell system allows generation of stable mammalian cell lines exhibiting tetracycline-inducible expression of a gene of interest from a specific genomic location (Ward et al., 2011). This *in vitro* system offers some advantages such as generation of isogenic, inducible and stable cell lines.

While regulation of NTCP gene and protein expression has been widely examined, (Anwer and Stieger, 2014; Appelman et al., 2021) kinase-mediated regulation of NTCP function is poorly understood. Most studies on NTCP post-translational regulation are based on rodent models. This is because of minimal NTCP expression in human hepatoma cell lines and downregulation of NTCP in primary human hepatocytes after isolation and depolarization (Yan et al., 2019; Appelman et al., 2021). Therefore, overexpressing cell lines with human NTCP expression or differentiated HuH-7 cells were employed to explore kinase-mediated NTCP regulation (Appelman et al., 2021; Saran et al., 2022a). Pretreatment with rapamycin reduced preS1 attachment by NTCP inhibition through a direct interaction with NTCP (Saso et al., 2018). Rapamycin analogs (rapalogs), everolimus and temsirolimus, inhibited HBV entry with higher potency (Saso et al., 2018). However, several macrocyclic peptides inhibited the NTCP-HBV interaction without affecting bile acid transport, making them attractive molecules for drug development (Passioura et al., 2018). In this brief study, several kinase inhibitors and mTOR inhibitors were evaluated for NTCP inhibition using taurocholate (TCA) as a probe bile acid substrate. Structurally similar mTOR inhibitors (everolimus, sirolimus and temsirolimus) and functional but structurally unrelated mTOR inhibitors (dactolisib and OSI-027) were examined to verify the direct interaction of NTCP with macrocyclic peptides.

## 2 Materials and methods

### 2.1 Chemicals, reagents, and antibodies

Kinase inhibitors were procured from various sources as shown in Supplementary Table S1. Ibrutinib, masitinib, neratinib, osimertinib, tucatinib, and vemurafenib were generously provided by the laboratory of Prof. Klarissa Jackson. [^3^H]-taurocholate (TCA; catalog number #NET322250UC, >97% radiochemical purity) was purchased from PerkinElmer, Inc. (Boston, MA). DMSO (#41639) and other reagents were obtained from Sigma-Aldrich. Primary antibodies against NTCP (#ab131084), GFP (#632592) and *ß*-actin (#A1978) were purchased from Abcam (Cambridge, MA), Takara Bio Clontech (San Jose, CA), and Sigma-Aldrich (St. Louis, MO), respectively. Secondary horseradish peroxidase (HRP)-conjugated goat anti-mouse (#115-035-003) and goat anti-rabbit (#111-035-144) antibodies were from Jackson ImmunoResearch (West Grove, PA).

### 2.2 DNA constructs

Plasmids containing GFP-tagged human NTCP (NM_003049) gene and empty vector were purchased commercially (GeneCopoeia, Rockville, MD). NTCP-GFP open reading frame was amplified using forward primer 5′- CTC​CTC​GGT​ACC​ATG​GAG​GCC​CAC​AAC​GCG​TCT-3′ and reverse primer 5′- CTC​CTC​GCG​GCC​GCT​CAC​TTG​TAC​AGC​TCG​TCC​ATG-3′ using PCR. Similarly, GFP open reading frame was amplified using forward primer 5′- CTC​CTA​GGT​ACC​ATG​GTG​AGC​AAG​GGC​GAG​G-3′ and reverse primer 5′- CTC​CTC​GGG​CCC​TCA​CTT​GTC​GTC​ATC​GTC​TTT​GT-3′. The following PCR program was used for amplification [denaturation at 98°C × 10s, annealing at 60°C × 30s, and extension at 72°C × 45s (NTCP-GFP), 30s (GFP) for 30 cycles]. A single PCR product (approximately 1.8 kb for NTCP-GFP and 0.8 kb for GFP) was visualized using a 1% agarose gel and ethidium bromide. An aliquot of the PCR products was purified and digested using high-fidelity restriction enzymes KpnI/NotI for NTCP-GFP and KpnI/ApaI (New England Biolabs, Ipswich, MA) for GFP. The pcDNA5/FRT/TO expression plasmid (Invitrogen), kindly provided by Prof. Bryan Roth and Dr. Justin English at UNC Chapel Hill, was used for ligation of the digested PCR products. Ligation was performed using T4 DNA ligase (#15224017, Invitrogen) at 16°C overnight. The reaction mix was heat-inactivated at 65°C for 10 min and then transformed into DH5α-competent *E. coli* cells (#18265017, Invitrogen, CA). The transformed bacteria were propagated with 1–5 µL of the ligation reaction mix or 5–10 ng of the *Flp* recombinase expression plasmid pOG44 (Invitrogen, CA) in super-optimal broth (S.O.C.; #B9020S, New England Biolabs) using the manufacturer’s protocol. Ampicillin-resistant bacterial colonies were selected and propagated overnight at 37°C in 5 mL of Luria-Bertani (LB) medium containing 200 μg/mL ampicillin with constant agitation at 250rpm using an orbital shaker. Plasmid DNA was isolated, purified using the QiaPrep Spin MiniPrep Kit (#27106, Qiagen), and screened by restriction digestion followed by agarose gel electrophoresis and Sanger sequencing (Eurofins Genomics, Louisville, KY).

### 2.3 Flp-In^™^ T-REx^™^ 293 cell culture

The Flp-In^™^ T-REx^™^ 293 cell line, derived from HEK293 cells, was generously provided by Prof. Bryan Roth. Cells were cultured in maintenance medium containing Dulbecco’s modified Eagle’s high-glucose medium (DMEM, #11965-092, Thermo Fisher Scientific), 10% fetal bovine serum (FBS; #F2442, Sigma-Aldrich), 100 U/ml penicillin, and 100 μg/mL streptomycin (#10378-016, Thermo Fisher Scientific). Prior to transfection, cells were also supplemented with 100 μg/mL Zeocin (#R25001, Invitrogen) and 5 μg/mL blasticidin S hydrochloride (#15205, Sigma-Aldrich) and maintained in a humidified incubator at 37°C and 5% CO_2_.

To generate Flp-In^™^ T-REx^™^ 293 cells with stable NTCP expression, cells were plated in 6-well dishes at 0.5 million cells per well in maintenance medium with Zeocin and blasticidin (day 0). The following day (day 1), cells were co-transfected with pOG44 and pcDNA5/FRT/TO/SLC10A1-GFP (NTCP-GFP) or pcDNA5/FRT/TO/GFP (Mock-GFP) plasmids in antibiotic-free maintenance medium. Transfection complexes were prepared by mixing 720 ng of pOG44 with 80 ng of pcDNA5/FRT/TO/SLC10A1-GFP or pcDNA5/FRT/TO/GFP in 50 µL of OptiMEM (51985-026, Thermo Fisher Scientific). Lastly, 2 µL of TransIT 2020 transfection reagent (#MIR5400, Mirus Bio) was diluted in 48 µL of OptiMEM, mixed with the DNA constructs in OptiMEM, and incubated at room temperature for 20 min. Following the 20-min incubation, the transfection complex (100 µL) was added to each well of Flp-In^™^ T-REx^™^ 293 cells and maintained in a humidified incubator at 37°C and 5% CO_2_. After 24 h of transfection (day 2), fresh maintenance medium containing the selection antibiotic hygromycin B (100 μg/mL; #10687010, Thermo Fisher Scientific) was introduced. Maintenance medium with hygromycin B was replaced every 2–3 days until visible foci were observed (14–20 days). Several visible foci for each cell line were selected and expanded into T25 and T75 flasks for further analyses, as described previously (Szczesny et al., 2018). The established cell lines expressing NTCP-GFP and Mock-GFP were cultured in maintenance medium containing 50 μg/mL hygromycin B. Cells were sub-cultured weekly and passages 4–15 were used for experiments.

### 2.4 HuH-7 cell culture

The human hepatoma HuH-7 cell line (JCRB0403) was purchased from Sekisui Xenotech (Kansas City, KS). Cells were cultured in maintenance medium [DMEM (#11995–065, Thermo Fisher Scientific), 10% FBS (#F2442, Sigma-Aldrich), 100 U/ml penicillin, and 100 μg/mL streptomycin (#10378–016, Thermo Fisher Scientific)]. The identity of the cell line was verified by amplification of 17 short tandem repeats by the UNC Vironomics Core.

Stable cell lines expressing NTCP-GFP and Mock-GFP were created using commercially available plasmids (GeneCopoeia, Rockville, MD). Briefly, NTCP-GFP (#EX-C0391-M98) and Mock-GFP (#EX-NEG-M98) encoding plasmids were transfected into HuH-7 cells using Lipofectamine 3000 (#L3000015, Invitrogen). HuH-7 cells were cultured in 100 mm dishes at 70% confluence prior to transfection. After 48 h of plating, cells were transfected with up to 2 μg DNA and Lipofectamine 3000 (1.5 μL per μg DNA), according to the manufacturer’s protocol. Transfected cells were incubated in a humidified incubator at 37°C and 5% CO_2_ for 48 h. Stably transfected cells were selected by adding 100 μg/mL geneticin (G418; #BP6735, Thermo Fisher Scientific) to the maintenance medium. Visible foci were isolated with the aid of cloning rings and subsequently cultured with 50 μg/mL G418.

Differentiated and undifferentiated HuH-7 were cultured as described previously (Saran et al., 2022a). Briefly, HuH-7 cells were seeded at 0.3–0.4 million cells per well in 24-well plates and the maintenance medium was supplemented with 1 μM dexamethasone (DEX) and 0.5% DMSO after 2 days to induce NTCP expression. Undifferentiated HuH-7 cells were cultured without medium supplements. HuH-7 cultures were overlaid with 0.25 mg/mL Matrigel Basement Membrane Matrix (#354234, Corning) in ice-cold maintenance medium with or without 1 μM DEX and 0.5% DMSO. Cells were maintained for two weeks with medium renewed every 2–3 days. Terminal assays were conducted after 14 days of culture and all experiments were performed between passage numbers 12–28.

### 2.5 Protein analysis

Doxycycline, another inducer for tetracycline-responsive promoters, was used to examine protein expression in NTCP-GFP and Mock-GFP Flp-In^™^ T-REx^™^ 293 cells. Cells were plated in poly-D-lysine-coated BioCoat^™^ 24-well plates (#08774124, Corning) at a seeding density of 0.5 million cells per well and allowed to adhere overnight. Doxycycline hyclate (#D9891, Sigma-Aldrich) was dissolved in water at 1 mg/mL, passed through a 0.22 μm filter and aliquoted prior to use. The following day, maintenance medium was replaced with maintenance medium containing doxycycline at 1 μg/mL. Cells were harvested 24 h after doxycycline addition. Briefly, cells were washed with ice-cold phosphate buffered saline (PBS) and lysed using RIPA buffer supplemented with protease inhibitors (#05892970001, Roche) and phosphatase inhibitors (#04906837001, Roche). After sonication (15 s), cell debris was separated using centrifugation (×17,000 g, 20 min, 4°C) and the supernatant was collected for Western blotting. Total protein in the whole cell lysates was determined using the Pierce BCA Protein Assay Kit.

Western blotting was performed using similar conditions as described previously (Saran et al., 2022b). Membranes were incubated overnight at 4°C with primary antibodies [anti-NTCP (1:1,000), anti-GFP (1:1,000), or anti-β-actin (1:10,000)] diluted in TBS-T with 5% BSA (w/v). Subsequently, membranes were incubated with HRP-conjugated goat anti-mouse or goat anti-rabbit secondary antibodies, diluted at 1:10,000 in blocking buffer, for 1 h at room temperature. Chemiluminescent signal was detected using SuperSignal West Femto Maximum Sensitivity Substrate and captured with a Molecular Imager VersaDoc imaging system (BioRad, Hercules, CA).

### 2.6 Immunofluorescence and imaging

Stably transfected Mock-GFP and NTCP-GFP HuH-7 cells were cultured at low density on glass cover slips on the bottom of 6-well plates (5-6 slips per well) in HuH-7 maintenance medium. Cells were cultured for three days and washed twice with PBS, followed by nuclear staining with Hoechst 33342. Subsequently, cells were washed twice with PBS, and GFP imaging was performed using a Zeiss LSM700 microscope (Zeiss, Göttingen, Germany) at an excitation wavelength of 488 nm and emission wavelength of 510 nm.

### 2.7 *In vitro* uptake studies in HuH-7 and Flp-In^™^ T-REx^™^ 293 cells

NTCP-GFP- and Mock-GFP-expressing HuH-7 cells were seeded at 0.3–0.4 million cells per well in 24-well plates (#353226, Corning) and cultured until fully confluent for three days. NTCP-GFP- and Mock-GFP-expressing Flp-In^™^ T-REx^™^ 293 cells were seeded at 0.3–0.4 million cells per well in poly-D-lysine-coated BioCoat^™^ 24-well plates. The following day after seeding, NTCP-GFP and Mock-GFP were induced using 1 μg/mL doxycycline for three days in Flp-In^™^ T-REx^™^ 293 cells. Uptake studies were performed in transfected HuH-7, differentiated HuH-7, and Flp-In^™^ T-REx^™^ 293 cells using [^3^H]-TCA, a probe bile acid substrate for NTCP. To evaluate drug-mediated inhibition of NTCP and [^3^H]-TCA accumulation, cells were incubated with either 0.1% DMSO (control) or 10 µM kinase inhibitors (Supplementary Table S1) for 15 min at 37°C prior to initiating [^3^H]-TCA accumulation. Troglitazone (75 µM) was used as a positive control for NTCP inhibition (Marion et al., 2007). Studies were performed using extracellular fluid (ECF) with and without sodium (Na^+^) ions. The composition of ECF buffer with Na^+^ was 122 mM sodium chloride, 25 mM potassium bicarbonate, 3 mM potassium chloride, 0.4 mM monopotassium phosphate, 10 mM D-glucose, 1.4 mM calcium chloride, 1.2 mM magnesium sulfate, and 10 mM HEPES, pH 7.4, whereas in the Na^+^-free ECF buffer, 122 mM sodium chloride was replaced with 122 mM choline chloride (Malinen et al., 2019a). HuH-7 and Flp-In^™^ T-REx^™^ 293 cells were washed twice with warm ECF buffer with Na^+^ or choline (Chol^+^). Subsequently, TCA accumulation was initiated for 10 min at 37°C using 2 µM [^3^H]-TCA (200 nCi/mL) in ECF buffer with Na^+^ or Chol^+^ in the presence of 0.1% DMSO control or 10 µM kinase inhibitors. Cells were washed twice with ice-cold ECF buffer with Na^+^. Plates were frozen at −20°C and processed further by lysis using 400 µL of 0.5% Triton X-100% and 0.005% Antifoam-A in PBS. Radioactivity of cell lysates was measured using Bio-Safe II counting cocktail (Research Products International Corp., Mt Prospect, IL) and a Tri-Carb 3100 TR liquid scintillation analyzer (PerkinElmer Inc.). The Pierce^™^ BCA Protein Assay Kit was used to determine total protein content per well, which was used to normalize TCA accumulation for each sample (*n* = 3). Na^+^-dependent TCA accumulation in NTCP-GFP-expressing Flp-In^™^ T-REx^™^ 293 cells was calculated by subtracting the accumulation in Chol^+^ ECF buffer from the accumulation in Na^+^ ECF buffer. All statistical analyses were performed using GraphPad Prism 7.03.

## 3 Results

### 3.1 Kinase inhibitor screening in NTCP-expressing HuH-7 cells revealed that everolimus decreased [^3^H]-TCA accumulation

**FIGURE 1:**
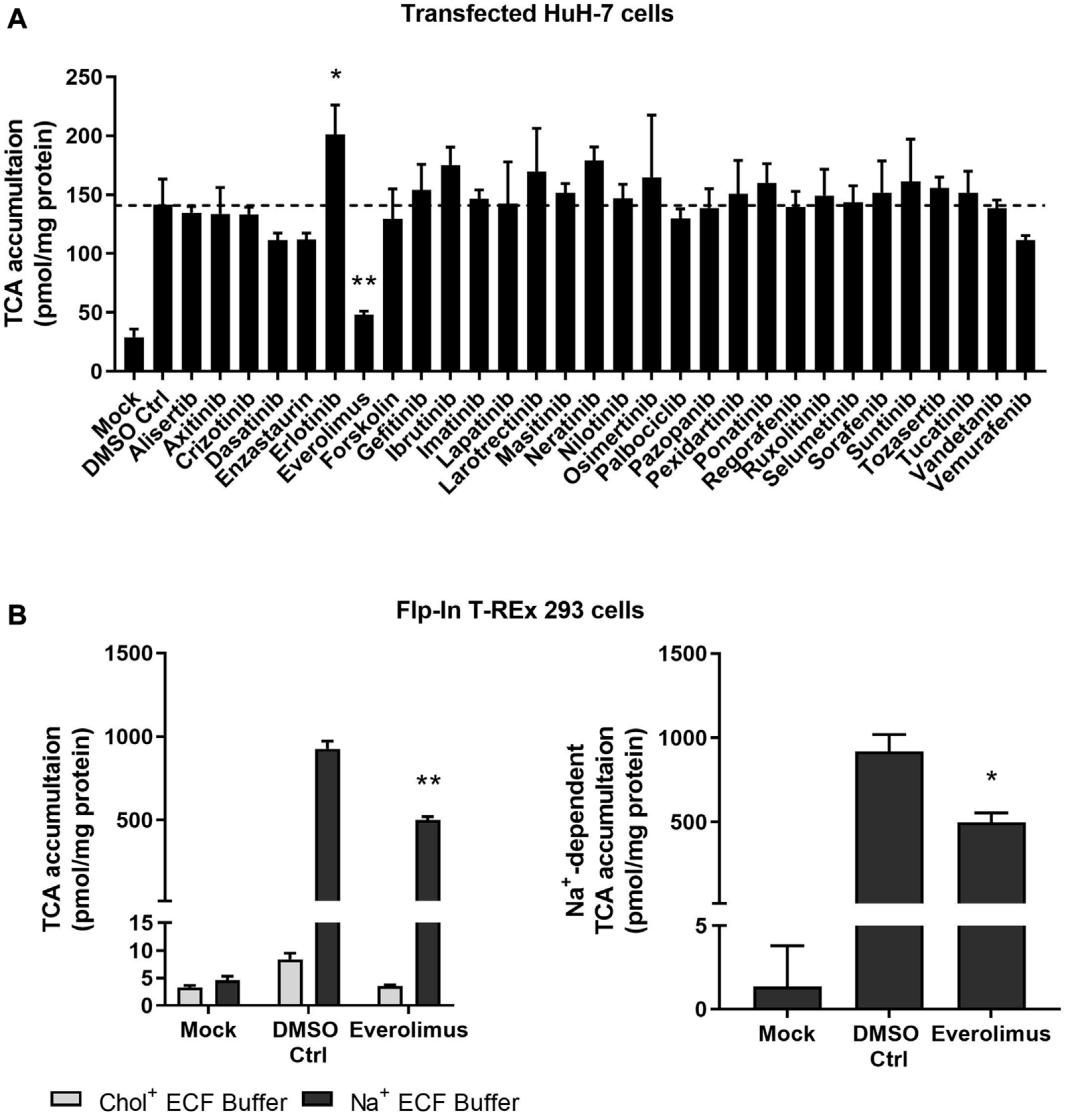
Effect of kinase inhibitors on NTCP-mediated transport of taurocholate (TCA). **(A)** Accumulation of [^3^H]-TCA (2 μM; 200 nCi/mL) in extracellular fluid buffer (ECF) with Na^+^ over 10 min was measured in Mock-GFP- and NTCP-GFP-expressing HuH-7 cells in the presence of 0.1% DMSO control or various kinase inhibitors (10 µM). NTCP-GFP-expressing HuH-7 cells were pre-incubated for 15 min with 0.1% DMSO control (DMSO Ctrl) or kinase inhibitors (10 µM) in cell culture medium prior to initiation of the accumulation phase. Data were plotted as mean ± SD (*n* = 3) and statistically significant differences compared to DMSO Ctrl were measured using an ordinary one-way ANOVA with Dunnett’s multiple comparison test (*, *p* < 0.05, **, *p* < 0.0001). **(B)** Mock-GFP or NTCP-GFP were induced in Flp-In^™^ T-REx^™^ 293 cells with 1 μg/mL doxycycline for three days and NTCP-GFP-expressing Flp-In^™^ T-REx^™^ 293 cells were pre-incubated with 0.1% DMSO control or 10 µM everolimus for 15 min in cell culture medium prior to initiation of the accumulation phase. Left panel: Accumulation of [^3^H]-TCA (2 μM; 200 nCi/mL) in ECF without Na^+^ (Chol^+^) or with Na^+^ over 10 min was measured in Mock-GFP- and NTCP-GFP-expressing Flp-In^™^ T-REx^™^ 293 cells in the presence of 0.1% DMSO control or 10 µM everolimus. Data were plotted as mean ± SEM (*n* = 3, in triplicate) Right panel: Na^+^-dependent [^3^H]-TCA accumulation was calculated based on the data in the left panel by subtracting [^3^H]-TCA accumulation in Chol^+^ buffer from [^3^H]-TCA accumulation in Na^+^ buffer. Data were plotted as mean ± SEM (*n* = 3, in triplicate). Statistically significant differences compared to DMSO Ctrl were measured using an ordinary two-way ANOVA with Dunnett’s multiple comparison test (**, *p* < 0.0001; left panel) or an unpaired two-tailed *t*-test (*, *p* < 0.01; right panel).

Localization and function of NTCP were verified in stably transfected HuH-7 cells. In Mock-GFP transfected cells, the GFP signal was primarily cytoplasmic while intense plasma membrane staining was observed in NTCP-GFP transfected HuH-7 cells (Supplementary Figure S1). Compared to un-transfected HuH-7 cells, NTCP-GFP transfected HuH-7 cells showed higher TCA accumulation in Na^+^ ECF buffer, confirming expression of NTCP (Supplementary Figure S1). To screen kinase inhibitors for NTCP inhibition, NTCP-expressing HuH-7 cells were utilized to measure effects on TCA accumulation by pre-treating cells with selected kinase inhibitors and co-exposing cells during TCA accumulation with selected kinase inhibitors. Compared with Mock-GFP transfected HuH-7 cells, NTCP-GFP-expressing cells displayed markedly higher TCA accumulation in Na^+^ ECF buffer (Figure 1A). Compared to DMSO control, everolimus significantly decreased TCA accumulation by ∼ 66% in Na^+^ ECF buffer (Figure 1A). Erlotinib increased TCA accumulation significantly, however, this effect was not observed for other epidermal growth factor receptor (EGFR) inhibitors tested including gefitinib, lapatinib, neratinib, osimertinib, and vandetanib. Other kinase inhibitors showed no significant modulation of NTCP function based on TCA accumulation in NTCP-expressing HuH-7 cells (Figure 1A).

### 3.2 NTCP protein was doxycycline-inducible and everolimus reduced NTCP-mediated [^3^H]-TCA accumulation in overexpressing Flp-In^™^ T-REx^™^ 293 cells

NTCP protein was induced in Flp-In^™^ T-REx^™^ 293 cells within 24–72 h after addition of 1 μg/mL doxycycline (Supplementary Figure S2). The effect of everolimus on NTCP was confirmed using NTCP-overexpressing Flp-In^™^ T-REx^™^ 293 cells. Like HuH-7 cells, NTCP-GFP-expressing Flp-In^™^ T-REx^™^ 293 cells showed greater TCA accumulation compared with Mock-GFP cells in Na^+^ ECF buffer (Figure 1B; left panel). However, TCA accumulation in NTCP-GFP-expressing Flp-In^™^ T-REx^™^ 293 cells was ∼6x higher than in transfected HuH-7 cells. In NTCP-GFP-expressing Flp-In^™^ T-REx^™^ 293 cells, the uptake ratio of TCA in Na^+^ ECF buffer compared to Na^+^-free ECF in the DMSO control was >110-fold (mean of 927 pmol/mg protein in Na^+^ ECF buffer vs. 8 pmol/mg protein in Na^+^-free ECF). Thus, almost all the TCA accumulation in Flp-In T-REx 293 cells may be attributed to Na^+^-dependent (NTCP-mediated) transport. Consistent with HuH-7 cells, everolimus in Flp-In^™^ T-REx^™^ 293 cells decreased Na^+^-dependent (i.e., NTCP-mediated) TCA accumulation significantly (∼46%), which was determined by subtracting TCA accumulation in Chol^+^ ECF buffer from TCA accumulation in Na^+^ ECF buffer (Figure 1B; right panel).

### 3.3 Inhibition of NTCP-mediated TCA accumulation by everolimus was concentration- and time-dependent

**FIGURE 2:**
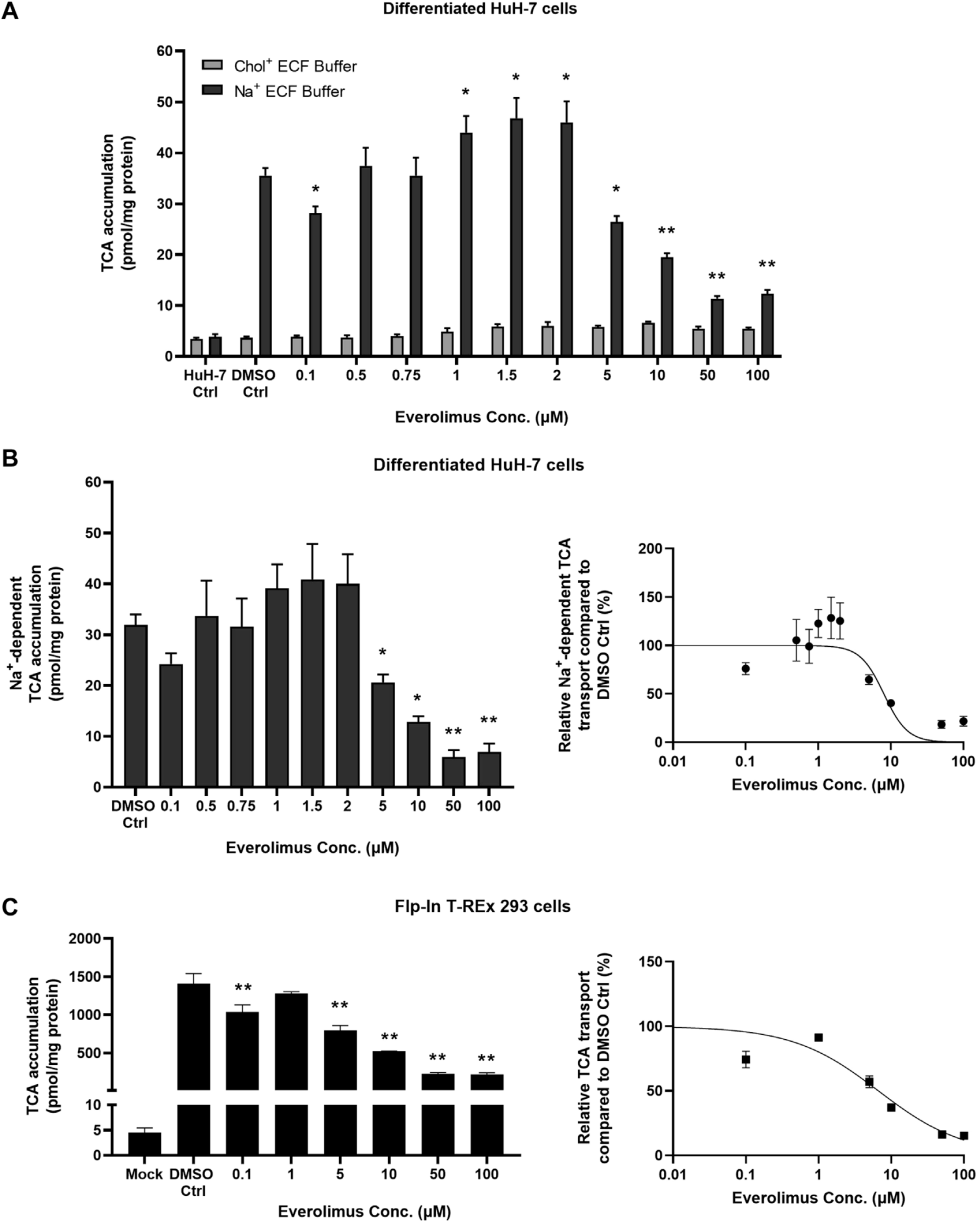
Concentration-dependent inhibition of NTCP-mediated taurocholate (TCA) transport by everolimus. Accumulation of [^3^H]-TCA (2 μM; 200 nCi/mL) in extracellular fluid buffer (ECF) with Na^+^ over 10 min in the absence (DMSO Ctrl; 0.1%) and presence of everolimus (0.1–100 µM) was measured in **(A)** undifferentiated HuH-7 cells (HuH-7 Ctrl) and differentiated HuH-7 cells. Data were plotted as mean ± SD (*n* = 3). Statistically significant differences compared to DMSO Ctrl were measured using an ordinary two-way ANOVA with Dunnett’s multiple comparison test (*, *p* < 0.05, **, *p* < 0.0001). **(B)** Left panel: Na^+^-dependent [^3^H]-TCA accumulation was calculated by subtracting [^3^H]-TCA accumulation in Chol^+^ buffer from [^3^H]-TCA accumulation in Na^+^ buffer. Right panel: Relative Na^+^-dependent [^3^H]-TCA transport was plotted with respect to everolimus concentration as a non-linear regression to calculate best fit IC_50_ of 8.0 ± 1.3 µM. **(C)** Left panel: Mock-GFP or NTCP-GFP were induced in Flp-In^™^ T-REx^™^ 293 cells with 1 μg/mL doxycycline for three days. Accumulation of [^3^H]-TCA (2 μM; 200 nCi/mL) in ECF with Na^+^ over 10 min in the absence (DMSO Ctrl; 0.1%) and presence of everolimus (0.1–100 µM) was measured in stably transfected Mock-GFP and NTCP-GFP-expressing Flp-In^™^ T-REx^™^ 293 cells. Right panel: Relative [^3^H]-TCA transport was plotted with respect to everolimus concentration as a non-linear regression to calculate best fit IC_50_ of 6.7 ± 1.3 µM. All data were plotted as mean ± SD (*n* = 3). Error bars are not visible when the SD is smaller than the data point. **(B)** and **(C)** Left panels: Statistically significant differences compared to DMSO Ctrl were measured using an ordinary one-way ANOVA with Dunnett's multiple comparison test (*, *p* < 0.05, **, *p* < 0.0001).

Concentration- and time-dependent inhibition of everolimus was examined without pre-treatment to differentiate between phosphorylation-dependent and direct NTCP inhibition. Differentiated HuH-7 cells cultured for two weeks expressed functional NTCP after addition of supplements (1 µM DEX, 0.5% DMSO and overlay) (Saran et al., 2022a). Undifferentiated HuH-7 cells (HuH-7 Ctrl) showed no difference in TCA accumulation between Na^+^ and Chol^+^ ECF buffers, indicating that functional NTCP was absent. In differentiated HuH-7 cells, the uptake ratio of TCA in Na^+^ ECF buffer compared to Na^+^-free ECF in the DMSO control was approximately 9-fold (mean of 36 pmol/mg protein with Na^+^ ECF buffer vs. 4 pmol/mg protein in Na^+^-free ECF; Figure 2A). In differentiated HuH-7 cells, everolimus inhibited TCA accumulation without pre-treatment in Na^+^-containing ECF buffer at 0.1 µM and at concentrations of 5 µM and higher, while TCA accumulation in Na^+^-free (Chol^+^) ECF buffer was unchanged compared to DMSO control (Figure 2A). A minor increase in Na^+^-dependent TCA accumulation was observed in differentiated HuH-7 cells incubated with 1–2 µM everolimus. Na^+^-dependent TCA accumulation decreased significantly at everolimus concentrations greater than 5 µM with an IC_50_ of 8.0 ± 1.3 µM (Hill slope = 2.7 ± 1.2; Figure 2B). Compared with Mock-GFP cells, TCA accumulation was >300-fold higher in NTCP-GFP expressing Flp-In^™^ T-REx^™^ 293 after induction with 1 μg/mL doxycycline for 72 h (Figure 2C). Consistent with differentiated HuH-7 cells, everolimus inhibited TCA accumulation at 0.1 µM and concentrations of 5 µM and higher using Na^+^-containing ECF buffer in NTCP-GFP expressing Flp-In^™^ T-REx^™^ 293 cells relative to Mock-GFP control cells (Figure 2C). The IC_50_ of everolimus for NTCP-mediated inhibition of TCA transport in Na^+^-containing ECF buffer was calculated as 6.7 ± 1.3 µM (Hill slope = 0.7 ± 0.1).

**FIGURE 3:**
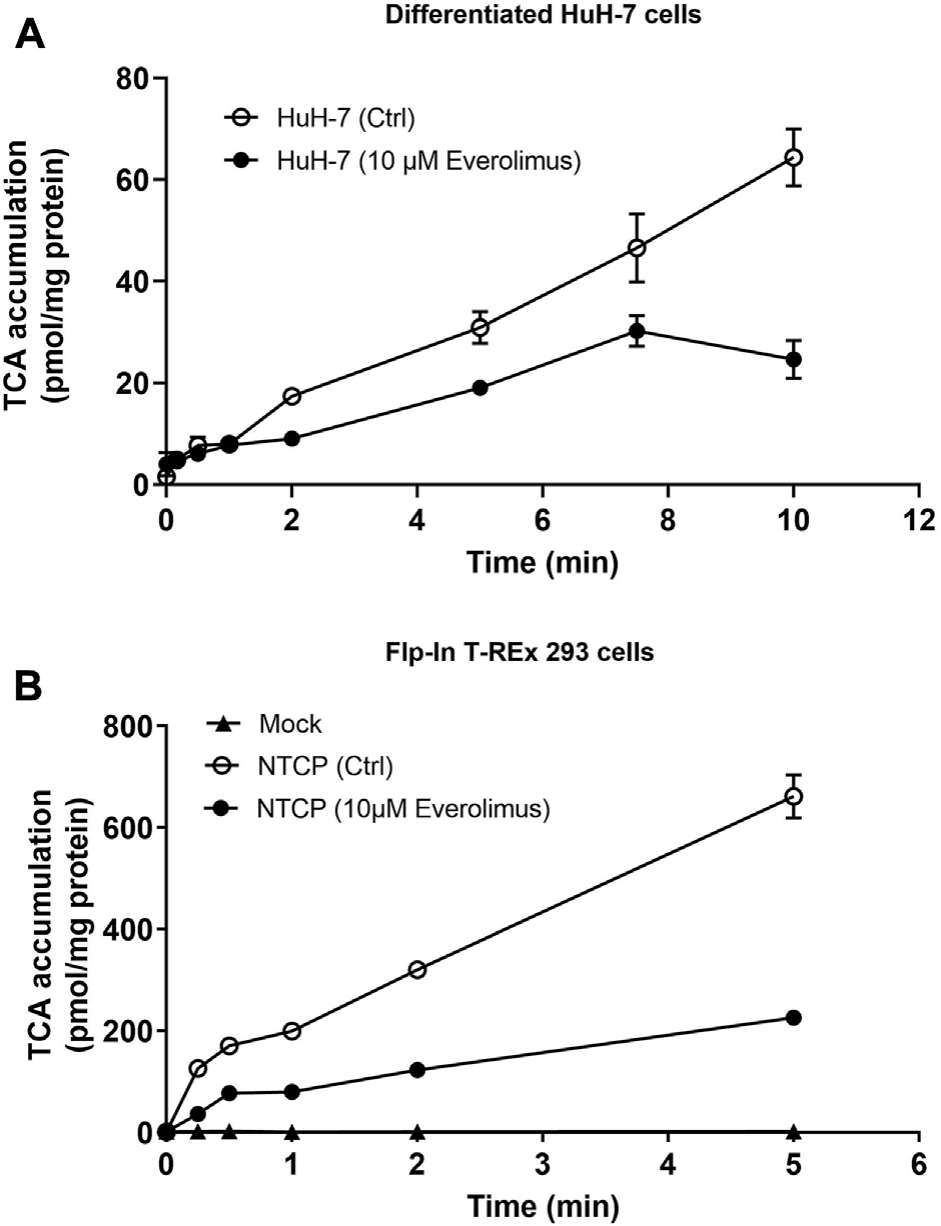
Inhibition of NTCP-mediated taurocholate (TCA) transport by everolimus over time. Accumulation of [^3^H]-TCA (2 μM; 200 nCi/mL) in extracellular fluid buffer (ECF) with Na^+^ over 5 or 10 min in the absence (DMSO Ctrl; 0.1%) and presence of everolimus (10 µM) was measured in **(A)** differentiated HuH-7 cells, and **(B)** stably transfected Mock-GFP and NTCP-GFP-expressing Flp-In^™^ T-REx^™^ 293 cells. Mock-GFP or NTCP-GFP were induced in Flp-In^™^ T-REx^™^ 293 cells with 1 μg/mL doxycycline for three days. All data were plotted as mean ± SD (*n* = 3). Error bars are not visible when the SD is smaller than the data point.

Everolimus (10 µM) inhibited TCA accumulation compared to DMSO control (Ctrl) over 10 min in differentiated HuH-7 cells without pre-treatment (Figure 3A). Inhibition was evident at 2 min and was >50% at 10 min; TCA accumulation decreased from 64 ± 6 pmol/mg protein in Ctrl to 25 ± 4 pmol/mg protein in the presence of 10 µM everolimus. Similarly, without pre-treatment, >50% inhibition was observed in NTCP-GFP expressing Flp-In^™^ T-REx^™^ 293 cells in the presence of 10 µM everolimus (226 ± 12 pmol/mg protein) compared to DMSO control (Ctrl; 662 ± 43 pmol/mg protein; Figure 3B). Inhibition was observed as early as 15 s in NTCP-GFP expressing Flp-In^™^ T-REx^™^ 293 cells, which suggests direct phosphorylation-independent NTCP inhibition in the absence of everolimus pre-treatment.

### 3.4 Inhibition of TCA accumulation by structural analogs but not functional analogs indicated direct NTCP inhibition

**FIGURE 4:**
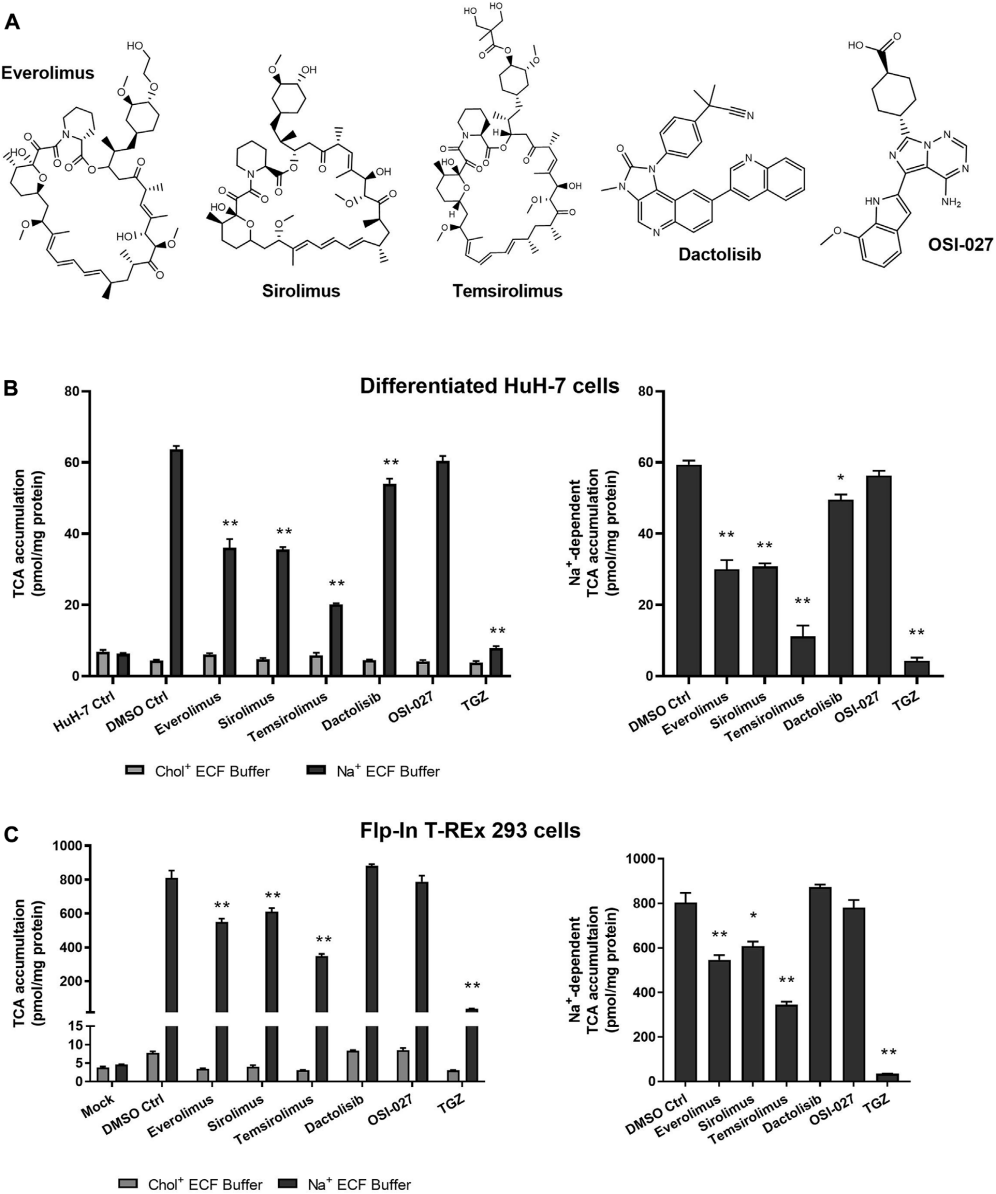
Effect of structural and functional analogs of everolimus on NTCP-mediated taurocholate (TCA) transport. **(A)** Structures of the mTOR inhibitors tested. Accumulation of [^3^H]-TCA (2 μM; 200 nCi/mL) in extracellular fluid buffer (ECF) with Na^+^ or without Na^+^ (Chol^+^) over 10 min was measured in the presence of 0.1% DMSO control (DMSO Ctrl) or mTOR inhibiting drugs (10 µM) in **(B)** undifferentiated (HuH-7 Ctrl) and differentiated HuH-7 cells, or **(C)** Mock-GFP and NTCP-GFP-expressing Flp-In^™^ T-REx^™^ 293 cells. Mock-GFP or NTCP-GFP were induced in Flp-In^™^ T-REx^™^ 293 cells with 1 μg/mL doxycycline for three days. All cells were pretreated for 15 min with 0.1% DMSO or drugs (10 µM) in cell culture medium prior to initiation of the accumulation phase. Troglitazone (TGZ; 75 μM), a known inhibitor of NTCP, was used as a positive control. **(B)** and **(C)** Left panels: Statistically significant differences compared to DMSO Ctrl were measured using an ordinary two-way ANOVA with Dunnett’s multiple comparison test (*, *p* < 0.05, **, *p* < 0.0001). **(B)** and **(C)** Right panels: Na^+^-dependent [^3^H]-TCA accumulation was calculated by subtracting [^3^H]-TCA accumulation in Chol^+^ buffer from [^3^H]-TCA accumulation in Na^+^ buffer. Statistically significant differences compared to DMSO Ctrl were measured using an ordinary one-way ANOVA with Dunnett’s multiple comparison test (*, *p* < 0.05, **, *p* < 0.0001). All data were plotted as mean ± SD (*n* = 3).

Sirolimus and temsirolimus were evaluated as structural analogs of everolimus (*i.e*., rapalogs). Dactolisib and OSI-027 were examined as functional analogs that inhibit mTOR but are structurally different from rapalogs (Figure 4A). Troglitazone (TGZ; 75 µM), a thiazolidinedione and a known inhibitor of NTCP, was used as a positive control. TGZ inhibited Na^+^-dependent TCA accumulation in differentiated HuH-7 and Flp-In^™^ T-Rex^™^ 293 cells by 93% and 96%, respectively (Figures 4B, C). In differentiated HuH-7 cells and NTCP-GFP expressing Flp-In^™^ T-REx^™^ 293 cells, everolimus, sirolimus and temsirolimus significantly inhibited TCA accumulation in Na^+^-containing ECF buffer and NTCP-mediated TCA accumulation (Figures 4B, C). While the decrease in Na^+^-dependent TCA accumulation by dactolisib was significant in differentiated HuH-7 cells, the effect was minor (15% decrease). Moreover, in NTCP-GFP expressing Flp-In^™^ T-REx^™^ 293 cells, dactolisib did not affect Na^+^-dependent TCA accumulation. Additionally, OSI-027 did not affect Na^+^-dependent TCA accumulation in differentiated HuH-7 cells or NTCP-GFP expressing Flp-In^™^ T-REx^™^ 293 cells. Therefore, only structural analogs of everolimus inhibited NTCP-mediated TCA accumulation, consistent with direct NTCP inhibition.

## 4 Discussion

This report reveals for the first time that everolimus, an immunosuppressive agent, inhibited NTCP-mediated TCA transport in a concentration- and time-dependent manner, consistent with direct inhibition of this primary hepatic bile acid uptake protein (IC_50_ 6.7–8.0 µM). Everolimus has been implicated previously as a drug that can induce cholestasis in humans, which could be attributed to potent inhibition of the human bile salt export pump (BSEP) even when NTCP-mediated bile acid uptake is impaired. Two patients with advanced metastatic renal cell carcinoma developed elevated blood γ-glutamyltransferase >3 times the upper limit of normal (ULN) and alkaline phosphatase >1.5 times ULN twenty days after initiation of treatment with everolimus (Pantano et al., 2010). In membrane vesicles prepared from Sf9 insect cells overexpressing human transport proteins, everolimus inhibited TCA transport by BSEP with an IC_50_ of 2 μM, and transport of estradiol-17β-D-glucuronide by the multidrug resistance-associated protein 2 (MRP2) with an IC_50_ of 11.3 µM (Morgan et al., 2013).

The functional impact of kinase inhibitors on NTCP was evaluated in this study to identify novel modulators of NTCP and explore potential kinase-mediated NTCP regulation. For this purpose, a lower, pharmacologically-relevant concentration of 10 μM was chosen for the inhibition screening, similar to previous reports (Donkers et al., 2017; Farasyn et al., 2019; Hayden et al., 2021). NTCP activity was largely unaffected by other kinase inhibitors in transfected HuH-7 cells, suggesting that NTCP function may not be kinase-dependent. However, this is yet to be confirmed in Flp-In^™^ T-REx^™^ 293 cells or human hepatocytes. The erlotinib-mediated increase in TCA accumulation may be due to inhibition of efflux transporters or NTCP stimulation; further studies are required to confirm the precise mechanism(s). Cell lines that expressed GFP-tagged NTCP were developed for this study using continuously cultured, undifferentiated HuH-7 cells and Flp-In^™^ T-REx^™^ 293 cells. Everolimus was identified as a NTCP inhibitor that significantly decreased [^3^H]-TCA accumulation in NTCP-expressing HuH-7 and Flp-In^™^ T-REx^™^ 293 cells. Both cell lines were used to confirm the everolimus-mediated effect on NTCP since HuH-7 cells also express organic anion transporting polypeptide (OATP) 1B1 and OATP1B3 (Malinen et al., 2019b). OATP1B1 and OATP1B3 are hepatic basolateral transport proteins responsible for bile acid uptake that are inhibited by everolimus (Farasyn et al., 2019). Consistent with the presence of OATP1B1 and OATP1B3, TCA accumulation in Mock-GFP-expressing HuH-7 cells was higher than in Mock-GFP-expressing Flp-In^™^ T-REx^™^ 293 cells (Figure 1). In contrast to HuH-7 cells, HEK293-derived cells show very low endogenous transporter expression (Ahlin et al., 2009). In differentiated HuH-7 cells (Figure 2A), TCA accumulation in control cells was lower than Mock-GFP transfected HuH-7 cells (Figure 1A) due to decreased OATP1B1 and OATP1B3 protein expression after differentiation (Saran et al., 2022a).

Previous reports indicated that rapamycin (sirolimus; 0.2 µM) did not inhibit TCA uptake in rat hepatocytes (Webster et al., 2000). This is consistent with results presented; the structural analog, everolimus, inhibited TCA uptake significantly at concentrations of 5 µM and higher, and sirolimus inhibited TCA uptake by 24%–48% at 10 µM. Interestingly, rapamycin (0.1 µM) was reported to decrease Ntcp mRNA in rat hepatocytes after a 24-hour treatment (Afroz et al., 2018). Further studies are needed to examine the effect of mTOR inhibitors on Ntcp/NTCP gene and protein expression. Additionally, cell viability of NTCP-expressing HepG2 cells was unchanged after a 2-hour treatment with various rapamycin derivatives, including everolimus, temsirolimus, and pimecrolimus at 64 µM (Saso et al., 2018).

Myrcludex B blocks HBV/HDV infection and inhibits NTCP-mediated bile acid uptake, suggesting that other NTCP inhibitors could potentially be used to treat HBV/HDV infection. This hypothesis was tested in NTCP-expressing U2OS cells and HepaRG^™^ cells with 1,280 compounds; five NTCP inhibitors (rosiglitazone, zafirlukast, TRIAC, Chicago sky blue 6B, and sulfasalazine) were identified that decreased bile acid uptake and blocked HBV/HDV infection *in vitro* (IC_50_ 5–10 µM). No rapalogs or mTOR inhibitors were tested in this study (Donkers et al., 2017). The present investigation demonstrates that everolimus reduced NTCP-mediated accumulation of the prototypical bile acid TCA. These findings identify an untapped chemical space that could be exploited through structure-activity relationship studies to develop more potent NTCP inhibitors (Kirstgen et al., 2021). The NTCP pharmacophore models suggest that two hydrophobic moieties and one hydrogen bond acceptor are important for NTCP inhibition (Dong et al., 2013; Dong et al., 2015). With recent structural elucidation of NTCP, (Goutam et al., 2022) new insights into the interaction of NTCP with mTOR inhibitors can be investigated and the underlying mechanisms of NTCP transport and inhibition could be leveraged to develop novel inhibitors for the treatment of liver diseases.

## Data Availability

The original data presented in the study are included in the article/Supplementary Material; further inquiries can be directed to the corresponding author.
