# Supplementary material for: Effect of mTOR inhibitors on sodium taurocholate cotransporting polypeptide (NTCP) function in vitro
Source: Front Pharmacol. 2023 Mar 22;14:1147495. doi: 10.3389/fphar.2023.1147495 (PMC10073475; doi:10.3389/fphar.2023.1147495)
Supplement: Supplementary file 1 [file DataSheet1.pdf]

Supplementary Material

Effect of mTOR Inhibitors on Sodium Taurocholate Cotransporting Polypeptide (NTCP) Function In Vitro

Chitra Saran[1,2†], Henry Ho[2†], Paavo Honkakoski[2,3], Kim L. R. Brouwer[2*]

[1]Department of Pharmacology, UNC School of Medicine, University of North Carolina, Chapel Hill, North Carolina, USA

[2]Division of Pharmacotherapy and Experimental Therapeutics, UNC Eshelman School of Pharmacy, University of North Carolina, Chapel Hill, North Carolina, USA

[3]School of Pharmacy, University of Eastern Finland, Kuopio, Finland

[†]These authors contributed equally to this work

Supplementary Table S1. Source and main targets of kinase inhibitors used to test inhibition of taurocholate (TCA) accumulation in NTCP overexpressing cells.

Kinase Inhibitor	Source (Catalog No.)	Main Targets
Alisertib	SelleckChem (S1133)	Aurora A
Axitinib	SelleckChem (S1005)	VEGFR1/2/3, PDGFRβ and c-Kit
Crizotinib	SelleckChem (S1068)	c-Met, ALK
Dasatinib	LC Laboratories (D3307)	Abl, Src, c-Kit
Gefitinib	LC Laboratories (G4408)	EGFR
Erlotinib	Cayman Chemical (10483)	EGFR
Everolimus	SelleckChem (S1120)	mTOR
Forskolin	SelleckChem (S2449)	PKA (adenylyl cyclase)
Enzastaurin	SelleckChem (S1055)	PKCβ
Ibrutinib	LC Laboratories (I3311)	BTK
Imatinib	Cayman Chemical (13139)	v-Abl, c-Kit, PDGFR
Lapatinib	SelleckChem (S2111)	EGFR, ErbB2
Larotrectinib	SelleckChem (S7960)	TRK
Masitinib	LC Laboratories (M7007)	c-Kit, PDGFRα/β
Neratinib	LC Laboratories (N6404)	HER2, EGFR
Nilotinib	Cayman Chemical (10010422)	Bcr-Abl
Osimertinib	LC Laboratories (O7200)	EGFR
Palbociclib	SelleckChem (S1116)	Cdk4/6
Pazopanib	SelleckChem (S3012)	VEGFR, PDGFR, FGFR, c-Kit, c-Fms
Pexidartinib	SelleckChem (S7818)	CSF-1R, c-Kit, FLT3
Ponatinib	SelleckChem (S1490)	Abl, PDGFRα, VEGFR2, FGFR1, Src
Regorafenib	Cayman Chemical (18498)	VEGFR1, PDGFRβ, c-Kit, c-RET, Raf-1
Ruxolitinib	Cayman Chemical (116095)	JAK1/2
Selumetinib	SelleckChem (S1008)	MEK
Sorafenib	Bayer HealthCare (R102403B)	VEGFR2/3, PDGFRβ, FLT3, c-Kit, Raf
Sunitinib	SelleckChem (S1042)	VEGFR2, PDGFRβ, c-Kit
Tozasertib	LC Laboratories (T2304)	Aurora A/B/C
Tucatinib	MedChem Express (HY16069)	ErbB2/HER2
Vandetanib	SelleckChem (S1046)	VEGFR2/3, EGFR
Vemurafenib	LC Laboratories (V2800)	B-Raf^{V600E}

ALK, anaplastic lymphoma kinase; BTK, bruton's tyrosine kinase; Cdk, cyclin dependent kinase; CSF-1R, colony stimulating factor 1 receptor; EGFR, epidermal growth factor receptor; FGFR, fibroblast growth factor receptor; HER2, human epidermal growth factor receptor 2; JAK, janus kinase; MEK, mitogen-activated protein kinase kinase; mTOR, mammalian target of rapamycin; PDGFR, platelet-derived growth factor receptor; PKA, protein kinase A; PKC, protein kinase C; TRK, tropomyosin receptor kinase; VEGFR; vascular endothelial growth factor receptor.

A

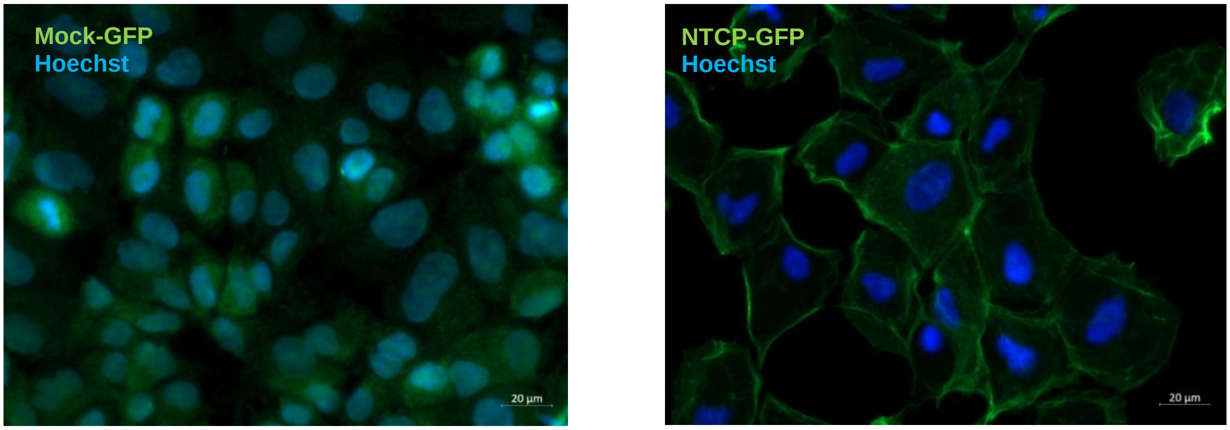

B

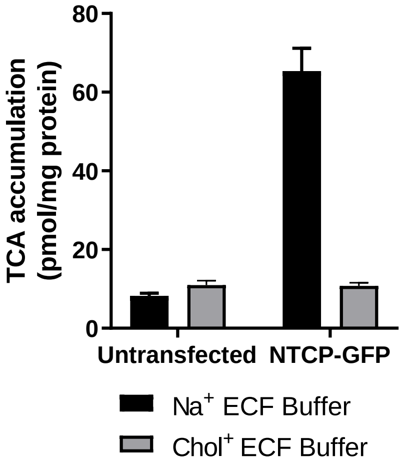

Supplementary Figure S1. A) Cellular localization of GFP in stably transfected Mock-GFP (left panel) and NTCP-GFP (right panel) HuH-7 cells. Immunofluorescence and bright field microscopy were performed to examine GFP localization. The nucleus was stained with Hoechst 33342 (scale bar = 20 µm). **B)** Accumulation of 2 µM [^{3}H]-TCA (200 nCi/mL) was measured in un-transfected control and NTCP-GFP expressing HuH-7 cells over 10 min. TCA accumulation was measured in extracellular (ECF) buffer with Na^+ or without Na^+ ($Chol^+$) in un-transfected and NTCP-GFP transfected HuH-7 cells after three days of culture. Data were plotted as mean ± SD (n=3).

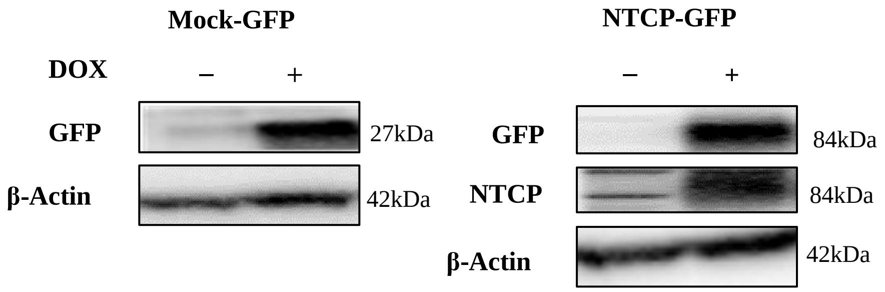

Supplementary Figure S2. Genes of interest were induced with the addition of 1 µg/mL doxycycline (DOX) to Mock-GFP and NTCP-GFP-expressing Flp-In™ T-REx™ 293 cells for 24 hours. Representative Western blot images with whole cell lysates to evaluate protein abundance of sodium taurocholate cotransporting polypeptide (NTCP), green fluorescent protein (GFP), and β-actin (loading control).
